# Influence of Metastatic Status and Number of Removed Lymph Nodes on Survival of Patients With Squamous Esophageal Carcinoma

**DOI:** 10.1097/MD.0000000000001973

**Published:** 2015-12-07

**Authors:** Feng Yuan, Zheng Qingfeng, Wang Jia, Lv Chao, Yan Shi, Wang Yuzhao, An Chao, Yang Yue

**Affiliations:** From the Department of Thoracic Surgery II, Laboratory of Carcinogenesis and Translational Research (Ministry of Education), Peking University School of Oncology, Beijing Cancer Hospital and Institute, Beijing, China.

## Abstract

The aim of this study was to determine the impact of lymph node (LN) metastasis conditions on the prognosis of patients with esophageal squamous carcinoma and the minimum number of LNs that should be removed to maximize overall postoperative survival among patients with this specific pathologic subtype.

In this study, 312 patients with thoracic squamous esophageal carcinoma who received in-patient thoracic surgery by the same surgeon in our hospital from August 1, 2003 to December 31, 2009 were recruited. Subsequently, Kaplan-Meier methods were used to determine associations between LN metastasis conditions and mortality and between the numbers of LNs removed during esophagectomy and mortality. Cox regression models were used to adjust for potential confounding covariates.

According to Kaplan-Meier analyses, the number of metastatic LNs was a good predictor for the prognosis of patients with esophageal squamous carcinoma and the dissection of ≥29 LNs during thoracic surgery significantly improved patient survival (*P* = 0.011).

Lymph node metastasis rates may be a significant predictor for the prognosis of patients with esophageal squamous carcinoma. The number of LNs removed during esophagectomy is an independent predictor for the survival of patients with esophageal squamous carcinoma with maximal postoperative survival after the removal of ≥29 LNs.

## INTRODUCTION

Esophageal cancer is currently the sixth highest cause of cancer-related mortality globally,^1^ and its incidence varies widely with 60-fold differences among high- and low-incidence regions.^2^ Esophageal cancer has its highest prevalence in China and is ranked fifth for morbidity and fourth for mortality in areas where the pathologic squamous carcinoma subtype is predominant.^3^

Among prognostic factors for patients with squamous esophageal carcinomas, lymph node (LN) metastasis is one of the most important factors. Accordingly, the 2010 American Joint Committee on Cancer (AJCC) staging system markedly changed the N classification of esophageal carcinoma to accommodate increased numbers of metastatic LNs as predictive of poor postoperative survival, and recommended lymphadenectomy to achieve more accurate N staging. Using data from our institution, we assessed the impact of LN metastasis conditions on prognosis among patients with esophageal squamous carcinoma, and determined the minimum number of LNs that should be removed to maximize overall postoperative survival among patients with this specific pathologic subtype.

## METHODS AND PATIENTS

### Inclusion and Exclusion Criteria

From August 2003 to December 2009, 369 patients were admitted to our hospital for esophageal cancers and received in-patient surgery by the same surgeon. Subsequently, 312 patients were recruited according to the following criteria (Table 1).

**TABLE 1:**
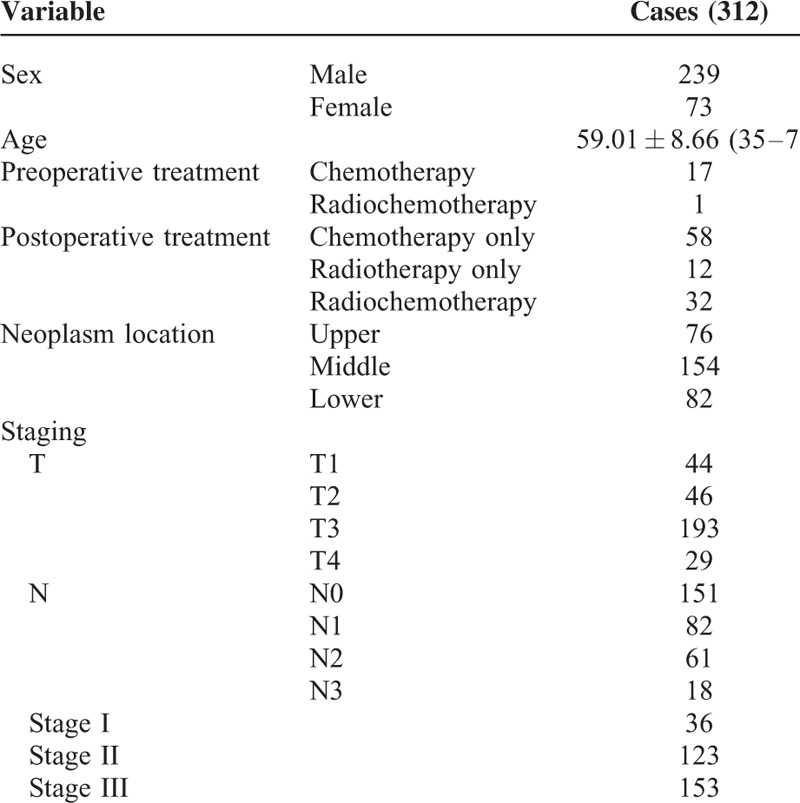
Status of the Patients Included

Eligibility criteria included primary esophageal squamous cell carcinoma tumor located in the thoracic esophagus, no distant metastasis, no LN larger than 1 cm around the celiac trunk or in the neck, and esophagectomy prescribed as a suitable curative surgical procedure.

Exclusion criteria included postoperative pathologic subtype other than squamous carcinoma, inoperable lesions even after neoadjuvant therapy (including invasion into the adjacent aorta, trachea, vertebra, or pulmonary structures), distant metastatic disease (including LN spread), no visible tumors after neoadjuvant therapy (chemotherapy or radiotherapy), or perioperative death.

### Surgical Procedures

Patients were treated using Sweet, Ivor-Lewis, or Mckeon esophagectomy^4–6^ during the study period according to tumor characteristics and the surgeon's preference.

All patients received double-lumen tracheal intubation and combined general and regional anesthesia with a thoracic epidural catheter. The dissection of intrathoracic esophagus included the en bloc resection of esophageal cancer and surrounding tissue. The proximal margin was at least 5 cm from the lesion and frozen sections were routinely obtained to secure tumor-free margins. The distal edging was 5 to 8 cm from the lesion according to tumor locations. All patients received 2-field (thoracic and abdominal) lymph-node dissection, and stations were defined according to American Thoracic Society criteria.

### Follow-up

Disease recurrence and survival was generally monitored at 1 month postoperatively, at 3-month intervals for 2 years, at 6-month intervals for the subsequent 3 years, and then at 1-year intervals. The last follow-up was performed in January 2012 and overall survival (OS) was calculated from the date of surgery to the date of death or last follow-up.

### Statistical Analysis

The Institutional Review Board at the Peking University Cancer Hospital approved this retrospective study and the requirement of patient consent was waived.

Overall survival was estimated using the Kaplan-Meier method and differences were compared using the log-rank test. Lymph node retrieval and staging were compared between 2 groups using Student *t* test or Mann-Whitney *U* test for analyses of normally or non-normally distributed data, respectively. Pearson χ^2^ test or the Fisher exact test were used to compare proportions as required and differences were considered significant when *P* < 0.05.

## RESULTS

### Prognostic Factors Identified Using Cox Regression Models

Cox regression analyses indicated that T and N classifications by the 2010 AJCC staging system, the numbers of dissected LNs, and the receipt of postoperative radiochemotherapy were significant predictors of patient survival.

### Prognostic Effects of Lymph Node Metastases

The current cases were divided into groups according to the numbers of LN metastases using the 2010 AJCC staging system as follows^7^: N0, no LNs metastasis, n = 151; N1, 1 to 2 LN metastases, n = 82; N2, 3 to 6 LNs metastasis, n = 61; and N3, 7 or more LN metastases, n = 18.

**FIGURE 1:**
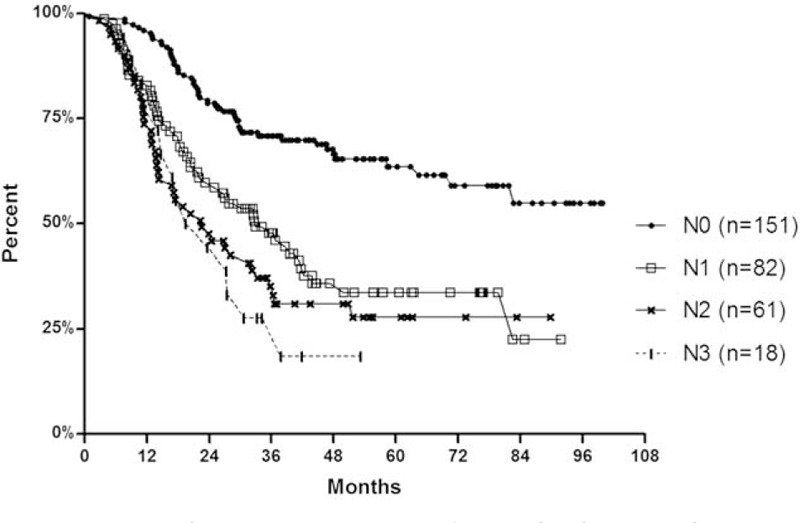
Kaplan-Meier estimates of mortality base on the number of lymph nodes involved. Significant difference is shown by Kaplan-Meier analyses between groups of patients with and without lymph node metastases (*P* < 0.001), whereas no significant differences were observed between N1, N2, and N3 groups (*P* = 0.312).

Kaplan-Meier analysis (Fig. 1) indicated significant differences between groups of patients with and without LN metastases (*P* < 0.001). No significant differences, however, were observed between N1, N2, and N3 groups (*P* = 0.312).

In further analyses, LN metastasis rates were calculated as numbers of metastatic LNs/numbers of dissected LNs, and prognostic factors were evaluated according to the staging criteria and patient groups shown in Table 2.

**TABLE 2:**
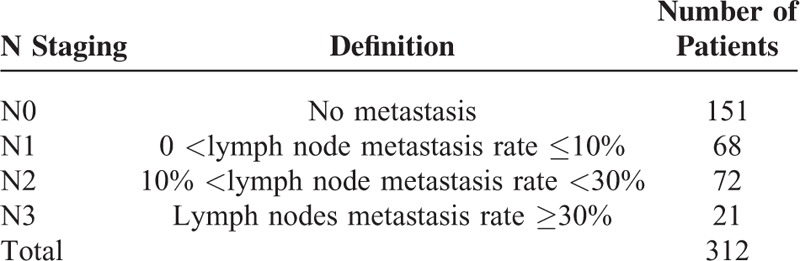
The Criterion of Node Staging by Lymph Node Metastasis Rate (= Number of Metastatic Lymph Nodes/Number of Dissected Lymph Nodes)

**FIGURE 2:**
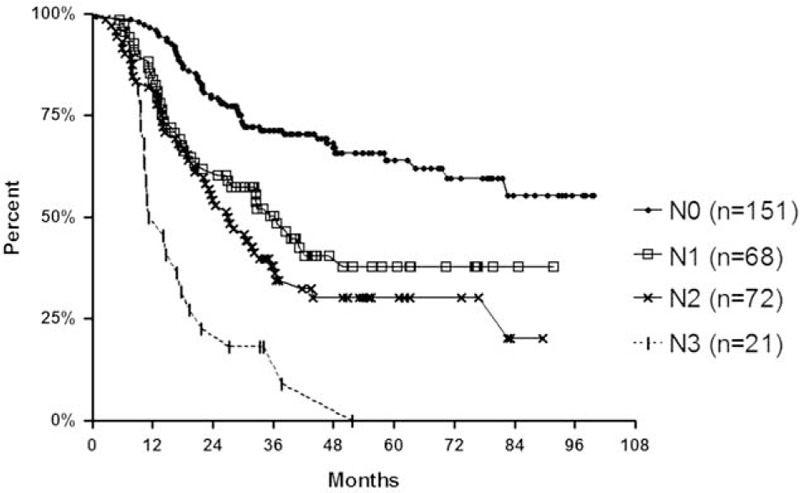
Kaplan-Meier estimates of mortality based on the rate of the lymph nodes involved. Significant differences are shown between all patient groups stratified by lymph node metastasis rates (*P* < 0.001).

Kaplan-Meier analyses indicated significant differences between all patient groups stratified by LN metastasis rates (Fig. 2; *P* < 0.001), and between N1, N2, and N3 groups (*P* = 0.001).

### Numbers of Dissected Lymph Node s and Prognosis

**FIGURE 3:**
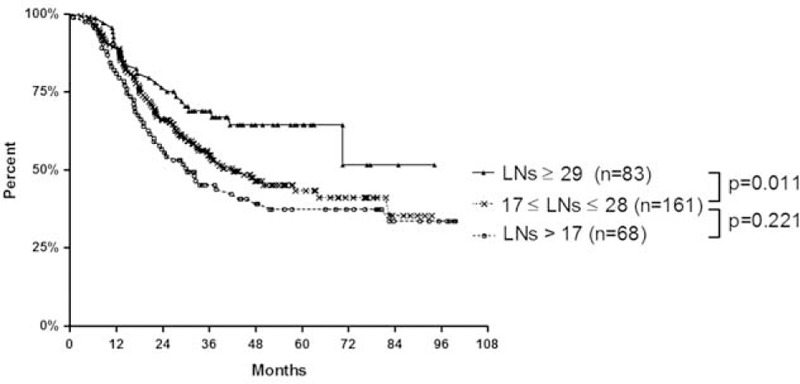
Kaplan-Meier analysis showed significant improvement survival rate with increasing number of dissected lymph nodes.

Cox regression analyses indicated that the number of dissected LNs is predictive of survival among patients with thoracic esophageal squamous carcinoma. Thus, patients were grouped according to the numbers of dissected LNs and comparisons of postoperative survival were made. Although, no effects were observed with the initial threshold of 10, the dissection of >17 LNs (thoracic plus abdominal field) significantly improved postoperative survival (*P* = 0.045). No further differences, however, were observed using higher thresholds (from 17 to 28) until comparisons were made with patients who underwent the removal of ≥29 LNs. At this threshold, postoperative survival was significantly improved (*P* = 0.011; Fig. 3)

To identify maximally prognostic criteria, patients were divided into 3 groups of <17, 17 to 28, and ≥29 removed LNs during esophagectomy. Subsequent Kaplan-Meier analyses showed significantly improved survival rates among patients who underwent the removal of >29 LNs (*P* = 0.018). The statistical difference exist only between group 17 ≤ LNs < 29 versus LNs ≥29 (*P* = 0.011), whereas *P* = 0.221 between group LNs < 17 and 17 ≤LNs < 29 (Fig. 3).

To assess >29 removed LNs as an independent prognostic factor, χ^2^ test were performed with pathologic staging and postoperative treatments as covariates, but no significant effects of these covariates were identified (*P* = 0.733 and *P* = 0.486, respectively; Table 3).

**TABLE 3:**
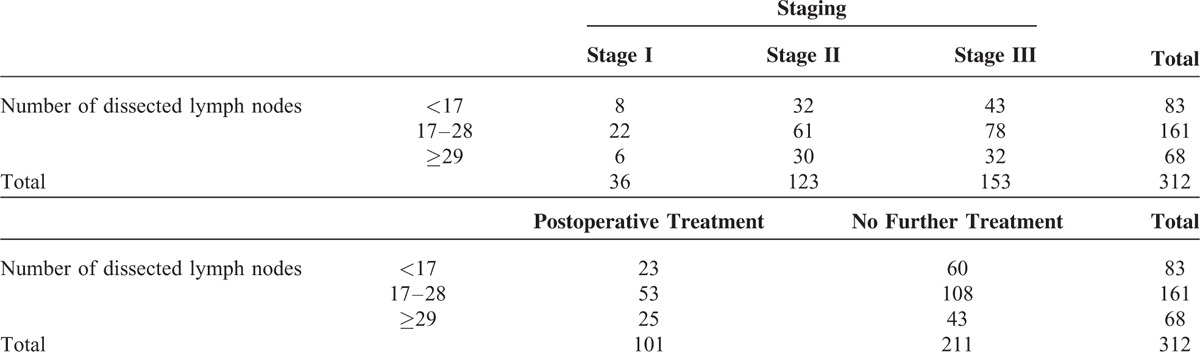
No Significant Effects of Pathologic Stage or Postoperative Treatment Were Found Between Patient Groups (χ^2^ test), Confirming that the Number of Dissected Lymph Nodes is an Independent Prognostic Factor for the Overall Survival of Thoracic Patients With Esophageal Squamous Carcinoma

## DISCUSSION

### Locations and Differentiation States of Neoplasms

In comparison with former editions, the 2010 AJCC staging system includes carcinoma locations and differentiation states as prognostic factors according to recent clinical studies. The current Cox regression analyses, however, eliminated these 2 factors, potentially reflecting the low rate of upper esophageal carcinomas (25% of patients). Hence, because the current staging system is based on both adenocarcinomas and squamous carcinomas of the esophagus, larger patient numbers may be required to demonstrate locations and differentiation states as significant prognostic factors.

### Lymph Node Metastasis

This revised 2010 AJCC staging classification is based on risk-adjusted random forest analyses of data from the Worldwide Esophageal Cancer Collaboration, including 13 institutions and 4627 patients who were treated with primary esophagectomy alone.^7^ This edition of N classification used absolute numbers of involved LNs as prognostic criteria. In agreement, Peyre et al^8^ demonstrated significant prognostic values of the numbers of LNs in 1053 patients from 9 esophageal centers. Although some other studies support further subdivision of absolute numbers of involved LNs,^9–12^ all previous analysis were based on data from combined cohorts of patients with squamous carcinomas and adenocarcinomas. In the current study, only patients with squamous carcinomas were included and N staging contributed significantly to the predictions of OS only for N0 and N1 groups.

In further analyses, the prognostic value of LN metastasis rates were assessed according to the numbers of metastatic LNs/numbers of dissected LNs. Previous studies also report use of LN metastasis rates using ratios of 0.1^13,14^−0.3^15^ or 0.2^16–18^ as decisive criteria, and in comparisons of patients with lymph metastasis rates lower and higher than 0.2, 5-year survival rates were decreased from 54% to 22%, and the risk of recurrence was increased from 44% to 59%.

In the current analyses, patients were divided into 4 groups (N0–N3) according to LN metastasis rates, and Kaplan-Meier analyses showed significant differences only between N1 to N3 groups. According to this result, we conclude that if dissected LNs are adequate for staging, LN metastasis rates may be more predictive of survival than absolute numbers of metastatic LNs. Hence, more a thorough dissection of LNs may lead to the identification of more normal and metastatic LNs; therefore, the improved accuracy of LN metastasis rates and higher prognostic value were achieved.

### Optimal Numbers of Dissected Lymph Nodes

The required number of LNs that should be dissected to achieve the best staging assessments and outcomes for patients with esophageal squamous carcinoma remains controversial. In agreement with previous studies, the numbers of pathologically examined LNs removed at the time of esophagectomy were significantly associated with prognosis in the current cohort^19^. Moreover, although the underlying mechanisms remain unknown, the dissection of ≥15 LNs is currently recommended by the National Comprehensive Cancer Network official guidelines for the surgical treatment of esophageal carcinoma.

Rizk et al^20^ analyzed 4627 patients from the Worldwide Esophageal Cancer Collaboration database, and suggested that the extent of lymphadenectomy was positively associated with survival among all patients with pN0 and pN(+) cancers, and recommended more LN dissections for patients with higher T status. Moreover, Groth et al^21^ used surveillance epidemiology and end results data from 4882 patients to determine the minimum number of LNs required to maximize survival, and indicated that the large numbers of LNs (30 or more) should be examined regardless of the surgical technique. Peyre et al^24^ also published a retrospective international multi-institutional study of 2303 patients who received R0 resections for esophageal carcinoma. These authors concluded that the number of removed LNs is an independent predictor of survival after esophagectomy for cancer, and a minimum of 23 regional LNs must be removed to achieve the maximum survival benefit. Finally, smaller studies also support this view,^22,23^ but the recommended thresholds vary between 12 and 30.

All of the previous studies on degrees of lymphadenectomy in esophageal cancer included both patients with adenocarcinoma and squamous carcinoma, and all report a preponderance of adenocarcinomas. Because the pathogenesis and biologic behaviors of these carcinomas may differ, further larger studies are required to examine prognostic factors in patients with only squamous carcinomas.

In summary, we analyzed survival rates among patients who underwent the dissection of 10 to 30 LNs, and used Kaplan-Meier analyses to show that postoperative survival differed between patients with <29 and ≥29 dissected LNs (*P* = 0.011).Cox proportional hazards models, however, failed to show other significant prognostic differences between these 2 groups. Hence, the number of removed LNs during esophagectomy is an independent predictor of survival among patients with esophageal squamous carcinoma, and >29 LNs should be dissected to maximize postoperative survival.
